# Enhancing ER stress in myeloma

**DOI:** 10.18632/aging.101273

**Published:** 2017-07-30

**Authors:** Craig T. Wallington-Beddoe, Stuart M. Pitson

**Affiliations:** Centre for Cancer Biology, University of South Australia and SA Pathology, Adelaide, Australia

**Keywords:** myeloma, ER stress, proteasome inhibitor, sphingolipids

Multiple myeloma (MM) is a haematological tumour of older people that to this day is still deemed incurable with a median overall survival of approximately 7 years despite recent advances in targeted therapies. The malignant plasma cells that constitute this tumour secrete monoclonal immunoglobulin that requires folding in the endoplasmic reticulum (ER) lumen. Even in normal plasma cells, this places a significant amount of stress on the ER chaperone and folding machinery which is heighted in MM due to sustained high production of monoclonal immunoglobulin. This ER stress activates the Unfolded Protein Reponse (UPR) which is a homeostatic process aiming to correct stress levels by globally slowing protein translation and increasing expression of ER chaperones and other folding machinery [1]. Critical to the amelioration of ER stress is the 26S proteasome which degrades proteins that cannot be correctly folded by the ER. Therefore, proteasome inhibitors are highly potent anti-MM agents since they prevent degradation of these misfolded proteins, producing augmented and sustained ER stress and activation of a terminal UPR that commits the MM cell to apoptosis [2].

The first-in-class reversible 26S proteasome inhibitor Bortezomib potently induces ER stress that results in a pro-apoptotic UPR and synergises with numerous chemotherapeutic agents [3]. Furthermore, the second generation, irreversible 26S proteasome inhibitor Carfilzomib is active in patients who are resistant to Bortezomib, as is Ixazomib, the first orally available 26S proteasome inhibitor [3]. Both are in advanced clinical trials and will enter the clinic in the near future. However, all of these agents induce ER stress by a similar mechanism, raising the question of whether alternate modes of inducing ER stress exhibit similar anti-MM efficacy and could potentially synergise with proteasome inhibitors. One such alternate mode of inducing ER stress involves altering the lipid composition of the ER membrane in favour of more saturated lipids, such as sphingolipids. Indeed, this strategy of increasing ER membrane lipid saturation induces activation of the UPR independent of the accumulation of ER lumenal misfolded proteins via the transmembrane domains of the ER stress sensors, protein kinase RNA-like endoplasmic reticulum kinase (PERK) and inositol-requiring enzyme 1 (IRE1) [4].

Sphingolipids are components of all cellular membranes with ceramide, a pro-apoptotic intermediary, central to sphiongolipid metabolism. *De novo* ceramide synthesis occurs at the ER membrane and perturbation of this process is hypothesised to alter ER membrane homeostasis, induce ER stress and activate the UPR without regard to lumenal misfolded protein burden. We have recently demonstrated that inhibition of the ER membrane resident enzyme sphingosine kinase 2 (SK2) induces ER stress and synergises with Bortezomib to target MM both *in vitro* and *in vivo* [5]. The observed synergy was associated with the accumulation of pro-apoptotic ceramide and marked increase in ER stress and UPR activation beyond that observed with either stress-inducing strategy alone. The pattern of UPR activation with SK2 inhibition was different from that observed with proteasome inhibitors, being consistent with ER membrane-initiated stress. Specifically, UPR activation was observed through PERK and IRE1 signalling, however, there was little increase in the lumenally-located ER chaperone (BiP), in contrast to that seen with Bortezomib where strong induction of BiP occured. Thus, sphingolipid modulation represents a very plausible therapeutic strategy for MM. However, the only agent currently in human clinical trials (https://clinicaltrials.gov/ct2/show/NCT02757326), for patients with relapsed/refractory MM, is Yeliva (ABC294640) which targets SK2 and dihydroceramide desaturase, both ER membrane enzymes [6]. Although this study is a phase I/II clinical trial that administers Yeliva alone without concomitant proteasome inhibition, the results are eagerly awaited, since they may provide the platform for a combinatorial approach that synergistically reduces MM disease burden in this subset of patients with few remaining therapeutic options.

Bortezomib is a component of many front-line chemotherapeutic regimens for newly-diagnosed MM, however, resistance to Bortezomib is a serious concern, eventually occurring in most patients [7]. The ability to re-sensitise patients to this highly effective agent is an important area of research as MM patients can be re-treated with Bortezomib if they have achieved at least a partial reponse to a Bortezomib-containing regimen at least 6 months previously, preferably in combination with a novel agent to achieve re-sensitisation [7]. Sphingolipid modulation is likely to find a place in this setting. Whilst there are several classes of therapeutic agents (immunomodulatory agents, histone deacetylase inhibitors, monoclonal antibodies, etc) that have been shown to produce such re-sensitisation to proteasome inhibitors in the relapsed/refractory setting [7], the combined use of agents that specifically heighten ER stress has hitherto not been examined. Thus agents that produce ER stress through disparate mechanisms from that of proteasome inhibitors could represent a significant advancement in MM pharmacotherapy with the development of sphingolipid enzyme modulators a potentially novel class of anti-MM agent.
